# High level of fluorescent oxidation products and worsening of asthma control over time

**DOI:** 10.1186/s12931-019-1173-0

**Published:** 2019-09-06

**Authors:** Zeina Akiki, Miora Andrianjafimasy, Farid Zerimech, Nicole Le Moual, Valérie Siroux, Orianne Dumas, Régis Matran, Rachel Nadif

**Affiliations:** 1 INSERM, U1168, Aging and chronic diseases. Epidemiological and Public health approaches, UMR-S 1168 Univ Versailles St-Quentin-en-Yvelines, F-94807, Villejuif, France; 2 Univ Versailles St-Quentin-en-Yvelines, UMR-S 1168, Versailles, France; 3 INSPECT-LB : Institut National de Santé Publique, Epidémiologie Clinique et Toxicologie, Faculty of Public Health, Lebanese University, Beirut, Lebanon; 4 CHU Lille, Service de Biochimie et Biologie moléculaire, F-59000, Lille, France; 5 EA4483, IMPECS, Institut Pasteur de Lille, Université de Lille, F-59000, Lille, France; 6 Institute for Advanced Biosciences, Centre de recherche UGA-Inserm U1209-CNRS UMR 5309, équipe d’épidémiologie environnementale, Site Santé, Allée des Alpes, F-38700, La Tronche, France; 7 CHRU de Lille, F-59000, Lille, France; 8 Univ Lille Nord de France, F-59000, Lille, France

**Keywords:** Fluorescent oxidation products, Oxidative stress, Adult asthma, Asthma control, Longitudinal study, Epidemiology

## Abstract

High Fluorescent oxidation products level (FlOPs), a global oxidative stress biomarker, was associated cross-sectionally with poor asthma outcomes but its longitudinal association with asthma evolution has never been examined. We aimed to study the associations between FlOPs level at baseline and changes in current asthma, asthma attacks and asthma control status over 8 years. We used data from the second survey of the French EGEA cohort study as baseline and the third survey as follow-up. At baseline, the mean age of the 489 participants with ever asthma was 39 (± 16) years, 49% were women. Among participants with controlled asthma at baseline, high FlOPs level was significantly associated with worsening of asthma control at follow-up (odds-ratio adjusted for age, sex and smoking status (95% CI): 2.27 (1.32–3.90). No other significant associations were observed. In conclusion, results suggest FlOPs as a predictor of asthma evolution in adults and a good candidate marker in asthma management.

*To the editor:*

Asthma is a chronic inflammatory disease of the airways with a strong clinical heterogeneity and phenotypic variability [1]. A recent approach recommends deconstructing asthma into component parts, in order to identify treatable traits [2]. In this context, integrating biological markers in asthma studies can open new possibilities for disease management [3, 4].

Along with inflammation, oxidative stress is an important pathogenic feature of asthma [5, 6]. Fluorescent oxidation products (FlOPs), a stable and easily measured biomarker of cumulative damages due to oxidative stress, was found to be associated with chronic diseases such as coronary heart disease (CHD) [7, 8] and chronic kidney diseases (CKD) [9]. In asthma, we recently reported that high FlOPs level was cross-sectionally associated with higher risk of asthma attacks and poor asthma control [10].

In the present letter, we further hypothesized that high FlOPs level was associated with asthma evolution, and investigated associations between plasma FlOPs level and changes in asthma characteristics over an 8 years period.

Data used for the analyses were collected in the framework of the Epidemiological Study on the Genetics and Environment of Asthma (EGEA) (https://egeanet.vjf.inserm.fr/), a French cohort study based on an initial group of asthma cases and their first-degree relatives, and population-based controls (EGEA1: 1991–1995, EGEA2: 2003–2007, and EGEA3: 2011–2013). The protocol and participant characteristics have been described previously [11, 12]. Ethical approval was obtained from the relevant institutional review board committees (Cochin Port-Royal Hospital and Necker-Enfants Malades Hospital, Paris). Participants signed a written informed consent.

We used data from the second survey (EGEA2) as baseline, and the third survey (EGEA3) as follow-up, and included adult participants with ever asthma at EGEA2 (*n* = 587) and followed-up at EGEA3 (*n* = 489). At both surveys, a total of 442 participants had available data on current asthma and 421 on asthma attacks. Among the 355 participants with current asthma at both surveys, 227 had available data on asthma control. Current asthma was defined by having had during the past 12 months: asthma attacks, or asthma symptoms (wheezing, nocturnal chest tightness, attack of shortness of breath), or medication use for breathing problems. Asthma control was assessed in 3 classes among participants with current asthma, by using responses to the EGEA2 and EGEA3 questionnaires to approximate closely the Global Initiative for Asthma 2015 definition [13]. Asthma control assessment was based on frequency of daytime symptoms, night waking due to asthma, use of reliever medication and activity limitations, assessed over the last 3-months [14]. For each participant, changes in current asthma, attacks, and control were categorized as “persistent” if the characteristic (current asthma, asthma attacks, or partly/uncontrolled asthma) was present at both EGEA2 and EGEA3, “improved” if the characteristic was present only at EGEA2, “worsened” if the characteristic was present only at EGEA3, and “stable” if the characteristic was not present at both surveys.

Plasma FlOPs level was measured at EGEA2 as described by Andrianjafimasy et al. [10] and according to Wu et al. [15]. FlOPs level was log-transformed due to its skewed distribution and expressed as geometric mean (GM) and Q1-Q3.

We studied the associations between FlOPs level and changes in “current asthma”, “asthma attacks”, or “asthma control” between EGEA2 and EGEA3. For each characteristic*,* we created two binary outcomes: 1) one outcome that compared the « persistent » to the « improved » group (reference) among participants who have the characteristic at EGEA2 (respectively with current asthma, with asthma attacks or with poor asthma control); 2) one outcome that compared the « worsened » to the « stable » group (reference) among those who did not have the characteristic at EGEA2 (respectively without current asthma, without asthma attacks or with controlled asthma). Multivariable logistic regressions were conducted using generalized estimating equations (GEEs) to take into account the familial dependence. In logistic regression models, we rescaled the value of FlOPs level using interquartile range (IQR) defined by the distance between the 25th and 75th percentiles, comparing an asthmatic with a “high” value of the FlOPs (in the middle of the upper half of the distribution) to an asthmatic with a ‘low’ value (in the middle of the lower half of the distribution) [16]. Odds-ratios were adjusted (aOR) for age (continuous), sex, and smoking status (never, ever-smokers). As a sensitivity analysis, ORs were also adjusted for pack-years instead of smoking status. The analyses were further repeated by excluding participants with history of CKD or CHD previously found to be associated with FlOPs [7–9]. Statistical analyses were performed using SAS software, version 9.4 (SAS Institute, Inc., Cary, North Carolina, USA). A *P*-value < 0.05 was considered statistically significant.

At EGEA2, the mean age of the 489 participants with ever asthma was 39 years (± 16.1), 49% were women, 52% were non-smokers, 85.5% had current asthma, 38% had asthma attacks (past 12 months), and 48.5% had poor asthma control (partly controlled/uncontrolled). The GM (Q1-Q3) for FlOPs level was 91.5 (78.6–102) RFU/mL. Participants included in the analyses (*n* = 489) had similar demographic and clinical characteristics (all *P* > 0.10), but were more non-smokers (*P* = 0.01) than participants lost to follow-up (*n* = 98).

Associations between FlOPs level at EGEA2 and evolution of asthma characteristics are shown in Table 1. Among participants with controlled asthma at EGEA2, high FlOPs level was significantly associated with worsening of asthma control at EGEA3 [_adjusted_OR (95% CI) = 2.27 (1.32–3.90)]. No other significant associations were observed. Adjustment for pack-years instead of smoking status or removing adults with history of CKD or CHD did not change the results (data not shown).

**Table 1:** Associations between Fluorescent oxidation products level at EGEA2 and evolution of asthma characteristics

Evolution of asthma characteristics	Fluorescent oxidation products (RFU/mL)		
	n	OR (95% CI)	OR_adjusted_ (95% CI)
*Persistent* vs *Improved (ref)*			
Current asthma^a^	355 vs 38	1.30 (0.86–1.98)	1.19 (0.79–1.80)
Asthma attacks^a^	115 vs 53	1.07 (0.74–1.53)	1.13 (0.74–1.72)
Poor asthma control^b^	82 vs 31	0.93 (0.58–1.47)	0.84 (0.48–1.44)
*Worsened* vs *Stable (ref)*			
Current asthma^a^	18 vs 31	0.95 (0.54–1.66)	0.73 (0.39–1.39)
Asthma attacks^a^	51 vs 202	1.10 (0.76–1.60)	1.08 (0.72–1.60)
Poor asthma control^b^	46 vs 68	**2.31** **(1.33–4.00)**	**2.27 (1.32–3.90)**

*OR* odds ratio expressed for an increase corresponding to the value of the interquartile range (distance between the 25th and 75th percentile) of FlOPs; adjusted for age, sex and smoking status. Results are presented as “Persistent *versus* Improved” (reference) and “Worsened *versus* Stable” (reference) between EGEA2 and EGEA3

^a^among 489 participants with ever asthma at both surveys; ^b^among 355 participants with current asthma at both surveys

Asthma attacks was defined by a positive answer to the following question: “*Have you had an asthma attack in the last 12 months?*”

Results in bold are statistically significant

To our knowledge, this study is the first to show that high FlOPs level is associated with worsening of asthma control over time. This result extends the association we previously reported in a cross-sectional setting [10]. The lack of association observed with current asthma was consistent with the result found in the cross-sectional design [10]. Furthermore, our results are in line with a recent review by Aldakheel et al. [17] drawing up an inventory of studies on biomarkers related to oxidative stress and asthma. Even if the longitudinal studies had small sample size (*n* < 40) and the asthma outcomes studied were asthma severity, atopic asthma or asthma treatments rather than asthma control, they reported significant associations between level of 8-isoprostanes or hydrogen peroxide and asthma outcomes.

No such consistency was observed for asthma attacks. One explanation may be that the definition of asthma attacks is not standardized and includes only one dimension of the disease. Larger studies are needed to clarify the specific dimensions of the disease for which FlOPs could be a relevant biomarker, and having FlOPs measurements at different points could help further examining the interest of monitoring this marker in the asthma management.

Previous prospective studies showed significant and positive associations between high FlOPs level and chronic inflammatory diseases such as the incidence of CHD among men without previous cardiovascular events, and the risk of future CHD in healthy women [7, 8]. Regarding asthma, the present study shows that high FlOPs level is associated with subsequent worsening of asthma control in adults and suggests that high FlOPs level could be a predictor of chronic inflammatory disease evolution. Although more studies and replications of our results are needed, it could be helpful to integrate FlOPs level in the approaches to identify “specific groups of patients” with controlled asthma who potentially may have bad prognosis.

## Data Availability

Due to third party restrictions, EGEA data are not publicly available. Please see the following URL for more information: https://egeanet.vjf.inserm.fr/index.php/en/contacts-en Interested researchers should contact egea.cohorte@inserm.fr with further questions regarding data access.

## Abbreviations

aOR: adjusted odds-ratio
CHD: Coronary heart disease
CKD: Chronic kidney diseases
EGEA: Epidemiological Study on the Genetics and Environment of Asthma
FlOPs: Fluorescent Oxidation Products
GEE: Generalized estimating equations
GM: Geometric Mean
IQR: Interquartile range
OR: Odds-ratio
Q1: First quartile
Q3: Third quartile
RFU: Relative Fluorescence Unit
